# Computational Prediction of Protein-Protein Interactions in *Leishmania* Predicted Proteomes

**DOI:** 10.1371/journal.pone.0051304

**Published:** 2012-12-10

**Authors:** Antonio M. Rezende, Edson L. Folador, Daniela de M. Resende, Jeronimo C. Ruiz

**Affiliations:** 1 Laboratório de Parasitologia Celular e Molecular, Centro de Pesquisa René Rachou – FIOCRUZ, Belo Horizonte, Minas Gerais, Brazil; 2 Departamento de Bioquímica e Imunologia, Instituto de Ciências Biológicas, Universidade Federal de Minas Gerais, Belo Horizonte, Minas Gerais, Brazil; 3 Instituto Oswaldo Cruz, Fundação Oswaldo Cruz, Rio de Janeiro, Rio de Janeiro, Brazil; 4 Laboratório de Pesquisas Clínicas, Universidade Federal de Ouro Preto, Ouro Preto, Minas Gerais, Brazil; Hospital for Sick Children, Canada

## Abstract

The Trypanosomatids parasites *Leishmania braziliensis*, *Leishmania major* and *Leishmania infantum* are important human pathogens. Despite of years of study and genome availability, effective vaccine has not been developed yet, and the chemotherapy is highly toxic. Therefore, it is clear just interdisciplinary integrated studies will have success in trying to search new targets for developing of vaccines and drugs. An essential part of this rationale is related to protein-protein interaction network (PPI) study which can provide a better understanding of complex protein interactions in biological system. Thus, we modeled PPIs for Trypanosomatids through computational methods using sequence comparison against public database of protein or domain interaction for interaction prediction (Interolog Mapping) and developed a dedicated combined system score to address the predictions robustness. The confidence evaluation of network prediction approach was addressed using gold standard positive and negative datasets and the AUC value obtained was 0.94. As result, 39,420, 43,531 and 45,235 interactions were predicted for *L. braziliensis*, *L. major* and *L. infantum* respectively. For each predicted network the top 20 proteins were ranked by MCC topological index. In addition, information related with immunological potential, degree of protein sequence conservation among orthologs and degree of identity compared to proteins of potential parasite hosts was integrated. This information integration provides a better understanding and usefulness of the predicted networks that can be valuable to select new potential biological targets for drug and vaccine development. Network modularity which is a key when one is interested in destabilizing the PPIs for drug or vaccine purposes along with multiple alignments of the predicted PPIs were performed revealing patterns associated with protein turnover. In addition, around 50% of hypothetical protein present in the networks received some degree of functional annotation which represents an important contribution since approximately 60% of *Leishmania* predicted proteomes has no predicted function.

## Introduction

According to the World Health Organization (www.who.int), there are roughly 12 million people infected with parasites from the *Leishmania* genus, which can cause visceral, cutaneous, or mucosal leishmaniasis [1], with an annual incidence from one to two million. Leishmaniasis is considered a neglected tropical disease responsible for a high estimated burden in Latin America [2].

For case control and the treatment of leishmaniasis, the major drugs used are either expensive, toxic, or both, and frequently require long periods of supervised therapy [2]. In addition, the pentavalent antimony based drugs that are the major chemical compounds used for leishmaniasis treatment have many side effects, such as pain, erythema, edema, abdominal pain, nausea, thrombocytopenia or leucopenia, and cardiotoxicity [1]. Furthermore, many reports of parasite resistance have been published [3]–[6]. It is worth mentioning that there are other medicines against leishmaniasis, but some of them are not economically feasible for many endemic countries [1].

To aggravate this situation, there are no effective vaccines for leishmaniasis. Despite abundant clinical and experimental evidence suggesting that leishmaniasis can be prevented by vaccination, the only proven vaccine agent in human beings is live *Leishmania major*, and it is discontinued because of unacceptable lesions in some recipients [1].

Therefore, based on the facts cited above, the necessity to develop new drugs and vaccine approaches is apparent. In order to reach this goal, new targets should be evaluated and the choice and evaluation method should consider the many different aspects of the complex biology of the agents of leishmaniasis. This challenging task can be achieved by integrating different data sets (e.g.; genome, transcriptome, proteome) in a systemic approach. Currently, this biology-based interdisciplinary approach focusing on the study of complex interactions in the biological system is called Systems Biology [7].

One of the main branches of Systems Biology refers to network studies. Here, there are different types of networks: protein-protein interaction network, metabolic network, regulatory network, etc. These networks can provide valuable information about different characteristics of an organism. More specifically, on a protein-protein interaction network (PPI) it represents a set of proteins of an organism, and how they interact with each other [8]. Moreover, the PPIs are undirected networks, in general are scale-free [9] and modular [10].

Currently, there are many different experimental methods to predict a PPI, among them we have the yeast two-hybrid method and affinity purification coupled with mass spectrometry [8]. Nevertheless, they may not be feasible for all proteins for all organisms, and they are susceptible to systematic errors. Thus, a number of computational approaches have been developed to predict protein-protein interactions based on protein or nucleotide sequence in large-scale [11]. Some of the computational approaches most known are the Phylogenetic Profile [12], [13], Genome Neighborhood [8], Gene Fusion [13], [14], Sequence Co-evolution [15], and comparison against the interaction public database or Interolog Mapping [16]–[19].

In this work, the Interolog Mapping method was used. Specifically on this approach, it assumes that if two proteins have a great sequence similarity against two proteins from a public database, and these latter ones interact, then the former ones interact too.

Therefore, the main point of this work is to predict a PPI network for each one of the target organisms, *Leishmania braziliensis*, *Leishmania major* and *Leishmania infantum*. Ultimately, we intend to use these networks to identify proteins and protein interactions that can be used as new targets for drugs and vaccines development.

## Methodology

### 1– Evaluation of PPI Prediction Approach

In order to evaluate the confidence of our network prediction methodology and consequently predict PPI networks for *Leishmania* sp, a performance evaluation was conducted.

The gold standard positive dataset was extracted from DIP (Database of Interacting Proteins) [7], [20]. The DIP database contains experimentally determined interactions between proteins, integrates information from many sources and is manually curated by experts. Given the information consistency of this database and taking into account the amount of information concerning PPI networks, *E.coli* was selected for the performance evaluation. Regarding the specific selection of positive pairs, we considered some points addressed on a recent work of Muley and Ranjan [21]. In this context, 702 interactions were selected as positive gold standard dataset.

The negative standard dataset used for the performance evaluation was built based on the works of [22]–[26]. In summary, considering all possible interactions in the model organism and subtracting the experimentally validated ones, a random selection was performed and only pairs containing proteins located in different subcellular localizations were maintained. A ratio of 1∶5 between positive and negative interaction pairs was used resulting into 3,510 negative interactions.

Using these gold standards datasets and the model organism we could identify the true positive (TP) or true negative (TN) protein pairs predicted by our network prediction methodology. The properly performance evaluation was made using ROC (Receiver Operating Characteristic) curves using the ROCR package for R (http://www.r-project.org/) [27]. A ROC curve is a plot of the False Positive Rate (FPR) against the True Positive Rate (TPR or sensitivity) for a given approach prediction. A random prediction will give value of 0.5 for the area under the ROC curve (or AUC) and a perfect prediction method would have an AUC value equal to 1 [21].

### 2– Data Filtering

Before starting with the network prediction, a filtering was performed on the predicted parasite proteomes to remove possible annotation errors. The proteome versions utilized here were version 2, final version, and version 3 for *L. braziliensis, L. major* and *L. infantum*, respectively. The following three criteria were utilized in this filtering. First, protein sequences should start with the methionine amino acid. Second, protein sequences should not have illegal characters such as X, B, Z, U, and “*” that are ambiguous or do not represent any of the 20 amino acids. Third, they should be bigger than 100 amino acids.

### 3– Predictions of Protein-Protein Interaction Pairs

To predict the protein-protein interaction pairs for the three organisms (*L. braziliensis*, *L. major* and *L. infantum*), the Interolog Mapping method was used. To apply the approach, we used four public databases namely: Domine [28], PSI-Base [29], IntAct [30], and String [31]. Here, it is worth mentioning the String database are not limited to direct, physical interactions between two proteins. Indirect interactions between proteins also exist such as genetic and metabolic interaction. Nevertheless, according to the last work describing the String database [32], most association currently can not be specified with much precision in terms of their mode of interaction. Thus the fundamental unit stored in String is the ‘functional association’. In addition, the String has flat files which have some degree of description about the interactions. If we consider in these files the term “binding” as physical interaction, we obtain nearly 94% of all interactions present in String. Besides, the other terms present in these files do not guarantee that the interactions are not physical interaction. Therefore, the impact of indirect interactions in our networks is minor. The first step here was to download all the interactions and all the protein sequences present in those databases. After that, the sequences from the predicted proteomes of the three protozoa were compared against the protein sequences from the databases and vice versa. To perform this comparison, we used the *blastp* from the BLAST software package [33] for searching sequences from PSI-Base, IntAct, and String. For the Domine database, the sequence comparisons were made by *hmmpfam* (sequence against HMM) and *hmmsearch* (HMM against sequence) from the HMMER software package [34], since the Domine uses the HMMs (Hidden Markov Models) present in the PFAM database [35] to describe its proteins.

Therefore, a protein *“X*” from a database is only considered as a homolog to protein *“A”* from one of the three organisms if protein *“X”* is the best hit for protein *“A”*, and protein *“A”* is the best hit for protein *“X”*. This is called the Best Bidirectional Hit (BBH). For each BBH, several measures were extracted. When a BBH came from *blastp* result, we extracted from it the minimum identity, minimum similarity and minimum alignment score between two sequences. In addition, the alignment coverage was extracted. When a BBH came from HMMER software, we extracted just minimum alignment score. In summary, the following formulas were applied:
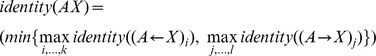


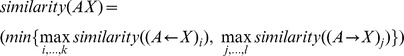


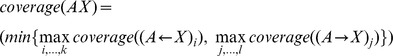


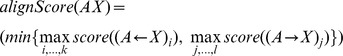



Here, *“A”* is a protein from a target organism, *“X”* is a protein from a database, *k* is the number of results from the comparison made using *“X”* as a query and *“l”* is the number of results from the comparison made using *“A”* as a query. From each comparison, the maximum values of each measure were taken. Afterward, just the minimum values from the two comparisons were used further. In addition, these measures were calculated for each database if the *e-value* for each comparison was smaller than 10^−85^ for String and IntAct results, 10^−45^ for Domine and 10^−10^ for PSI-Base.

We then mapped the interactions present in the databases on the three proteomes. To do that, we firstly knew that “*X”* and “*Y”*, which are proteins from a database, interact. Second, we knew that “*X”* was the BBH for “*A”,* which is a protein from a target organism, and “*Y”* was the BBH for “*B”,* which is also a protein from the same target organism of *“A”* protein. Hence, we assumed that *A* and *B* interact.

In general each database has a confidence score for its interactions, thus these scores were used to compose the final combined score. In our case, we were not able to find this kind of score for PSI-Base repository. In the end, for each database, the score for each prediction was calculated according to the followings:



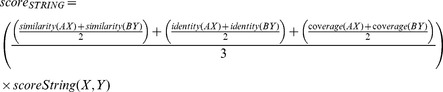


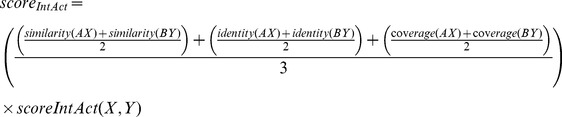


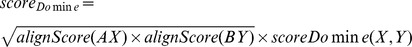


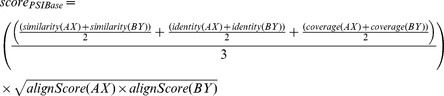



### 4– Calculating Confidence Score for Protein-Protein Interaction

In order to attribute a confidence score for the predicted interactions, we adopted the same rational described by [16], [36] and built a dedicated interaction combined score for our methodology. This combined score takes into account the prediction scores obtained from Domine, PSI-Base, IntAct and String Databases from each protein interaction and is calculated according to the formula shown below:
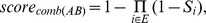
where *score_comb(AB)* is the combined score for the interaction between proteins “*A”* and “*B”*, *E* is all the methods that were used to predict the interactions, and *S_i_* is the score normalized by the biggest value calculated for the method *i*.

Many observed networks fall into the class of scale-free networks, meaning that they have power-law (or scale-free) degree distributions and this does not occur with random networks. Thus, after the calculation of *score_comb*, the three predicted PPIs were tested against the scale-free model for PPIs suggested by Barabasi and Oltvai [37] and the hierarchical model suggested by Ravasz *et al* [10]. The evaluation was made using Network Analyzer Version 2.7 [38] plug-in at Cytoscape Version 2.8.3 [39], [40]. Besides, our networks had their Clustering Coefficient and Mean Shortest Path compared against 1,000 random networks produced by Random Network Version 1.0 (http://sites.google.com/site/ randomnetworkplugin/) plug-in at Cytoscape. For that, the empirical *P-values* were calculated.

### 5– Gene Ontology Annotation

For the functional annotation attribution we adopted the classification vocabulary defined by the Gene Ontology Consortium [41] (GO - http://www.geneontology.org/). The ontology covers three domains: cellular component, the parts of a cell or its extracellular environment; molecular function, the elemental activities of a gene product at the molecular level, such as binding or catalysis; and biological process, operations or sets of molecular events with a defined beginning and end, pertinent to the functioning of integrated living units: cells, tissues, organs, and organisms.

The GO annotation schema adopted in this work came from the public Kinetoplastid database TriTrypDB version 4.1 (http://tritrypdb.org/tritrypdb/) [42]. This database provides for each of three GO ontologies two kinds of evidence of annotation, one is called annotated and the other predicted. In order to guarantee a higher confidence on the functional annotation, when possible the annotated terms were used for further analysis.

### 6– Predicting Functional and Conserved Modules

At this part of the work, our goal was to identify functional modules that are conserved in the predicted networks. Functional modules can be understood as a group of proteins functionally or physically linked that work together to reach a distinct function [43]. Moreover, according to Ravasz *et al* [10], PPIs in general have a modular or hierarchical architecture.

Then, to perform this prediction, we chose the networkBLAST program [44] that performs two basic tasks: a) the comparison of multiple PPI networks; and b) the prediction of functional modules. The algorithm also combines interactions along with sequence information in order to produce a network alignment graph. Each node in this graph defines a group of similar proteins whereas links between nodes defines putative complexes that are evolutionarily conserved across the three predicted networks. Interactions reliability scores are assigned using a probabilistic model and the similarity information necessary for that is obtained from a comparison of all versus all sequences present in the predicted PPI networks.

Afterwards, in order to characterize the clusters or modules found, a functional annotation schema is required. As described in an earlier section, the GO functional annotation was used.

Following the functional annotation described above and considering the Biological Process ontology, a GO term enrichment analysis was performed using the GO::TermFinder [45] for each cluster. In this approach, the statistical significance is determined using the hypergeometric distribution to calculate the *P-*value:
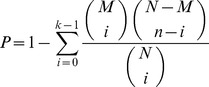
here, 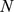 is equal to total number of proteins in the background distribution, which is the number of proteins in a PPI network that received at least one GO term, *M* represents the number of proteins within that background distribution that are annotated (either directly or indirectly) to any GO term of interest. *n* is the size of the list of proteins of interest (in our case it is the number of proteins in the module of interest). Finally, *k* is the number of proteins within that list or module which are annotated to the GO term of interest. Besides, as we were dealing with multiple hypotheses test, a correction for each *P-*value should be applied. Here, GO::TermFinder applied the Bonferroni correction.

### 7– Topological Analysis

The metrics used in order to extract biological information from the predicted PPIs were calculated using the CytoHubba Version 1.6 plug-in [46] at Cytoscape. In this work we used Degree and Maximal Centrality Clique (MCC). According to CytoHubba developer site (http://hub.iis.sinica.edu.tw/cytoHubba/supplementary/index.htm), the MCC topological index showed the highest overlap with known essential proteins of PPI network of S*accharomyces cerevisiae.* The reported overlap was 80% for the top 10 proteins and 70% for the top 100 proteins of the network. Considering this outstanding performance, we use the MCC index to rank the top 20 proteins from the three predicted PPI network.

Moreover, the variability of the top ranked proteins was also assessed based on the ortholog group information present in the TriTrypDB. In this database, the proteins of the Kinetoplastids are clustered in groups based on OrthoMCL database information (http://www.orthomcl.org/cgi-bin/ OrthoMclWeb.cgi). Thus, for each ortholog group associated with the top ranked proteins a multiple sequence alignment was performed using MAFFT [47] and the mean identity evaluated with the *alistat* program from HMMER package.

Complementarily, the immunologic potential of the selected top ranked proteins was addressed using BCPred12 [48], which is a predictor for potential epitopes recognized by B cells, NetCTL [49] and NetMHCII [50] which are predictors for potential epitopes with affinity binding to MHC class I and II alleles respectively. Finally, the predicted proteomes of *Mus musculus* (mouse), *Canis lupus familiaris* (dog) and *Homo sapiens* (human) were downloaded from NCBI repositories (http://www.ncbi.nlm.nih.gov/) on August 24, 2012 and used to address the sequence similarity between these genomes and the top 20 proteins ranked by MCC.

### 8– Evolutionary Analysis

It has been described that the proteins with high Degree (the degree of a node in a network is the number of connections it has to other nodes) probably are proteins more conserved and ancient [51]–[53]. Then, in order to assess this assertion, we compared Degree and the nucleotide diversity index (π) [54] of the proteins present in the predicted PPI networks. This measure was obtained first by defining a Degree range in the predicted networks. The ranges were 2 to 10, 11 to 20, 21 to 30, 31 to 40, 41 to 50, and greater than 50. The selected proteins jointly with their orthologs extracted from the TriTrypDB were aligned using MAFFT, and then the π was calculated for each ortholog group using the Variscan program [55].

### 9– Hypothetical Proteins Analysis

In the strict sense, hypothetical proteins are defined as proteins computational predicted from nucleic acid sequences that have not been shown to exist by any experimental evidence. Furthermore, these proteins are characterized by low identity to the known annotated proteins in public domain databases.

The term “conserved hypothetical proteins” is also broadly employed and describes a fraction of genes in sequenced genomes that are found in organisms from several phylogenetic lineages but that have not been functionally characterized and described at the protein chemical level.

Trypanosomatids genomes are known to have a large amount of hypothetical proteins (∼60%) [56], [57], and these might be involved in essential cellular processes. Therefore, due to the importance and amount of these proteins in the genomes that we are working with and the possibility to use the PPI network to infer a function for them, we decided to apply an approach called FS-Weight [58] to try to obtain a clue on the possible functions for the hypothetical proteins.

The FS-Weight method, which stands for Functional Similarity Weight, is based on direct and indirect functional association using PPI networks as the main input. Either direct or indirect neighbors of a protein may share some physical or biochemistry features that allow them to bind to this protein. Therefore, this method has as an advantage that it does not use only direct interaction partners, which would limit prediction to proteins that have at least one interaction partner with known annotation, actually, FS-Weight also uses indirect interaction partners which increases the chance of predicting a protein function [58]. Furthermore, it calculates a functional similarity between two proteins, not necessarily from direct partners, based on the topological context of both proteins and the reliability of the interactions they do. This calculation is applied in order to reduce the effects of including erroneous interactions. Hence, the more common proteins exist interacting with two any proteins the chances that these two proteins share some biological function are higher. In addition, FS-Weight gives greater weight to common neighbors than non-common ones [58]. It is also worth mentioning that the FS-Weight performance was not re-evaluated for Leishmania species. The work that described the approach utilized data of *S. cerevisae* to validate the method. Therefore, some caution must be taken in using the function predictions made for hypothetical proteins present in our networks.

Moreover, to apply this annotation approach it is necessary to use an annotation schema that has already been used for the proteins with known functions. For this purpose we used the three GO ontologies already described.

## Results

### 1– Evaluation of PPI Prediction Approach

As detailed at Methods section, in order to evaluate the confidence of our network prediction methodology, gold standards positive and negative datasets were built from DIP database using the protein interaction data from *E.coli*, used here as model organism. This high quality control dataset that integrates 702 positive protein pairs and 3,510 negative protein pairs was used in the performance evaluation made by Receiver Operating Characteristics (ROC) graphs.

**Figure 1:**
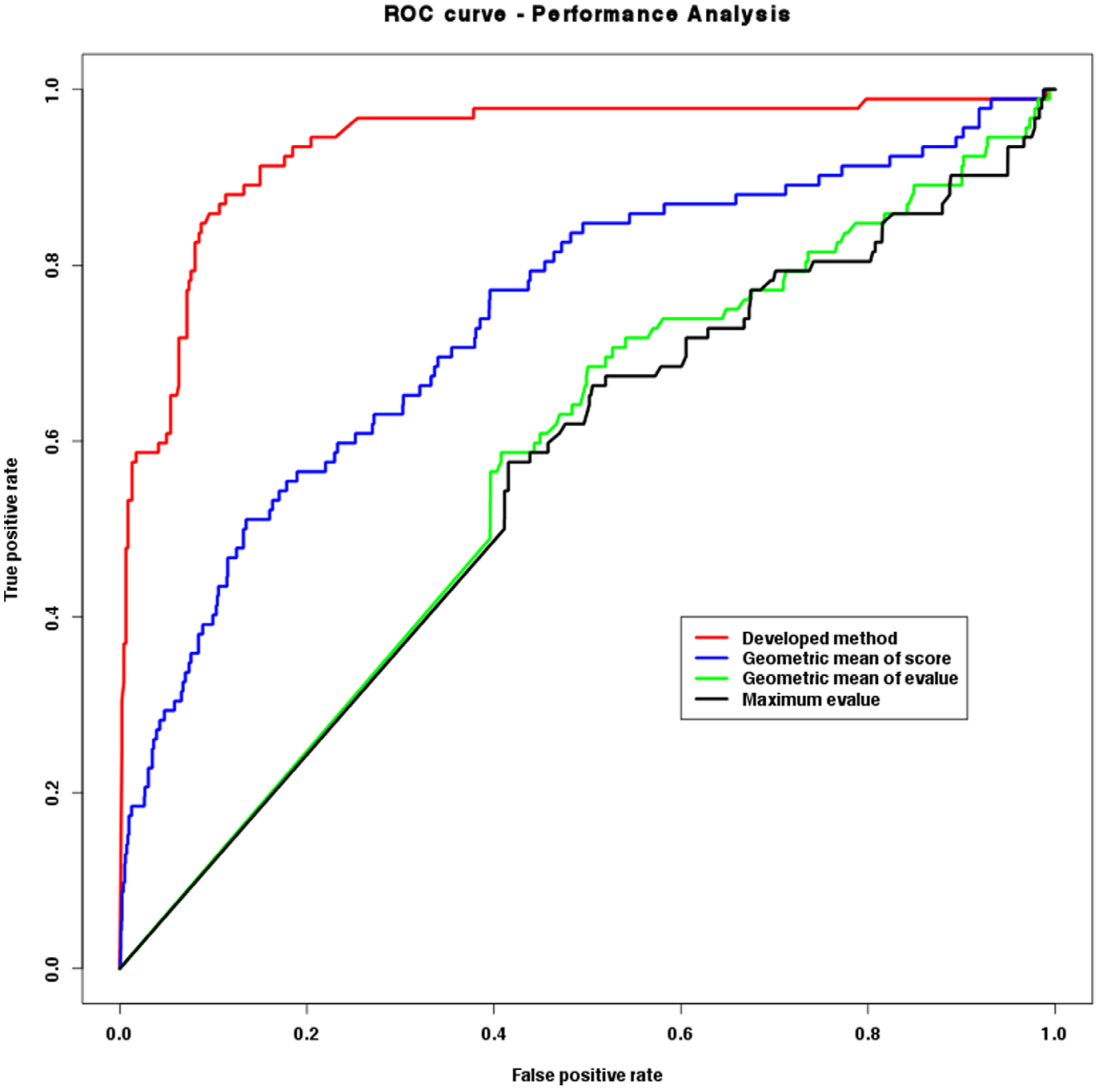
Performance evaluation of approached used to predict PPI networks using the ROC curve. Here the predictions were compared against a gold standard data of interactions extracted from DIP database for *E. coli* (see text for details).

The accuracy of the proposed methodology measured by the area under the ROC curve can be addressed on Table 1 and through the plot presented on Figure 1. The AUC value of 0.94 obtained for *score_comb* indicates the robustness of the approach adopted. However, this result should be considered carefully since the databases used for the interolog-mapping contain many *E. coli* interactions. This might lead the evaluation of the confidence of our networks to some degree of bias.

**Table 1:** Performance evaluation of approach used to predict PPI networks.

Measure of confidence	AUC value
Developed method	0.94
Geometric mean of score	0.74
Geometric mean of *evalue*	0.57
Maximum *evalue*	0.55

### 2– Filtering of Data and PPIs Prediction

As mentioned earlier, a filtering step was performed on the three proteomes in study in order to select sequences that were correctly annotated. A small percentage of proteins were excluded from our analyses since they presented possible errors. Then, the predicted proteome of *L. braziliensis*, *L. major* and *L. infantum* lost 4.33%, 2.95%, and 4.78% of proteins, respectively (Table 2).

**Table 2:** Number of proteins in the predicted proteome of the target organisms before and after the filtering.

Organism	Total of proteins	Total of proteins after filtering	Relative number of lost proteins (%)
*L. braziliensis*	8310	7950	4.33
*L. major*	8408	8160	2.95
*L. infantum*	8216	7823	4.78

Subsequent to the filtering process, the three proteomes were used for PPIs prediction. These predictions were made based on different databases, such as Domine, PSI-Base, IntAct and String. Using these evidences, we proposed and calculated a combined score for the interactions predicted in the PPIs which ranged from 0 to 1. Afterwards, it was possible to demonstrate the wellness of fit of scale-free models for the three predicted PPIs (Table 3).

**Table 3:** Fitting results for scale-free model, and Clustering Coefficient and Mean Shortest Path for PPIs compared against the same measure extracted from 1000 Random PPIs.

*Leishmania braziliensis*			
Scale free model	Correlation	R^2^	
	0.941	0.816	
Random model			
Measure	Modeled PPI	Random PPIs	P-value
Clustering Coefficient	0.433	0.159±0,003	p<0.05
Mean Shortest Path	2.877	2.579±0,004	p<0.05
***Leishmania major***			
Scale free model	Correlation	R^2^	
	0.925	0.815	
Random model			
Measure	Modeled PPI	Random PPIs	P-value
Clustering Coefficient	0.430	0.157±0.003	p<0.05
Mean Shortest Path	2.914	2.584±0.004	p<0.05
***Leishmania infantum***			
Scale free model	Correlation	R^2^	
	0.940	0.829	
Random model			
Measure	Modeled PPI	Random PPIs	P-value
Clustering Coefficient	0.424	0.160±0.003	p<0.05
Mean Shortest Path	2.886	2.573±0.004	p<0.05

The PPIs predicted were then compared against 1,000 random networks. The Clustering Coefficient and the Mean Shortest Path were compared (Table 3). The values of the Clustering Coefficient of the PPIs are much greater than the random networks adding an extra layer of credibility for the predicted networks.

**Figure 2:**
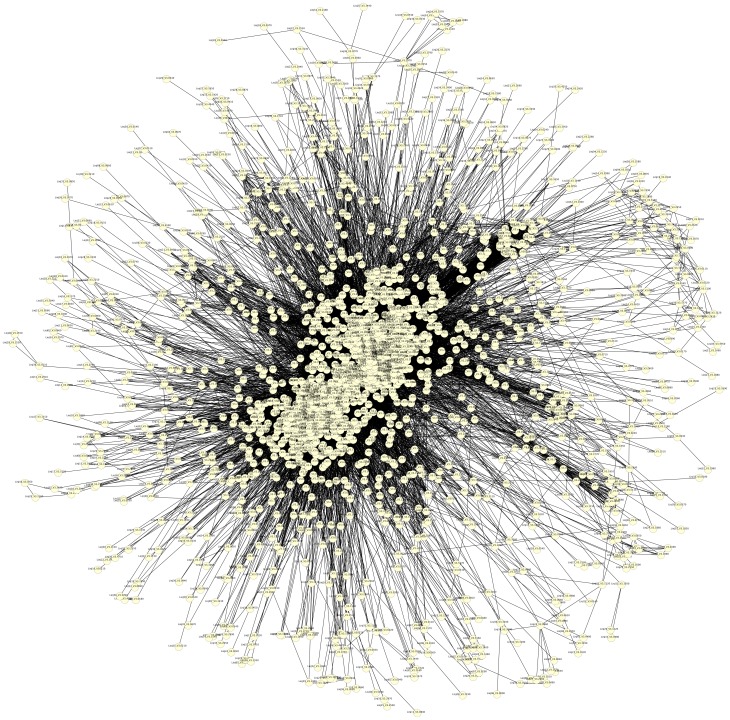
Protein-Protein Interaction for *L. infantum* visualized using Cytoscape 2.8.3 and the Edge-weighted spring embedded layout.

As a result, the predicted PPIs incorporated 23%, 24%, and 25% (Table 4) of the proteins from the filtered proteomes of *L. braziliensis* (Table S1), *L. major* (Table S2) and *L. infantum* (Table S3) respectively. Figure 2 shows one of the three networks.

Furthermore, we used GO terms to try to draw a function profile of the networks. For this analysis, we used the predicted terms present in TritrypDB database instead of annotated terms. The rationale underlying this choice was associated with the small number of GO terms annotated for *L. braziliensis* that would prevent its comparison against the other two leishmanias. The three ontologies (Biological Process, Cellular Component, and Molecular Function) were applied and similar results were found. Considering a frequency larger than 2 for a given GO term, it is worth pointing out that the total intersection among the predicted networks was 79%, 84%, and 75% for Biological Process, Cellular Component, and Molecular Function, respectively. In fact, from the top 10 most frequent GO terms for each ontology, 8 of them for Biological Process, 7 of them for Molecular Function and all of them for Cellular Component are the same for the three networks.

**Table 4:** General features of the three predicted PPI Networks.

Organism	Number of Nodes (Proteins)	Number of Interactions	Number of hypothetical protein	Number of hypothetical protein annotated (%)*
*L. braziliensis*	1818	39420	381	153 (40%)
*L. major*	1947	43531	416	200 (48%)
*L. infantum*	1959	45235	415	229 (55%)

* Proteins were annotated following the methodology described in the text.

### 3– Evolution Analysis

**Figure 3:**
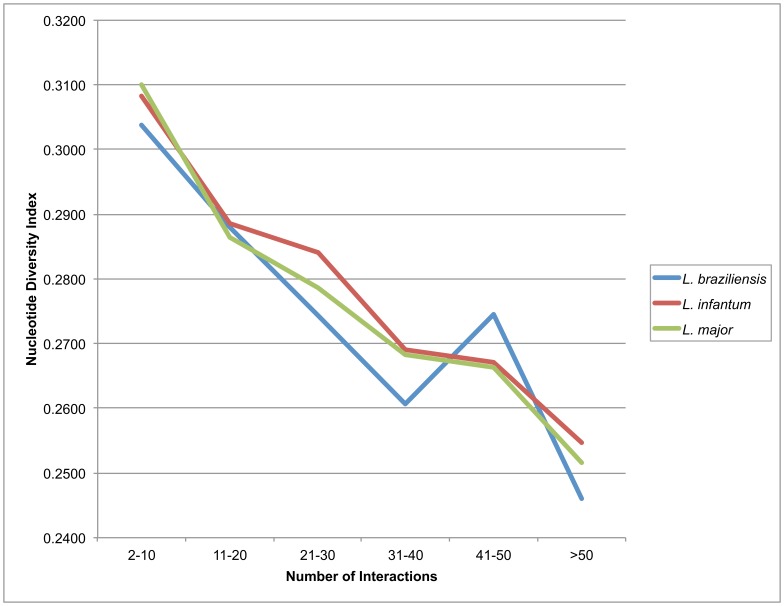
Degree versus diversity analysis. Graph of median of Nucleotide Diversity (π) measure (Y axis) versus Degree range (X axis) of three PPIs.

In order to obtain information relative to the correlation between the number of interactions that a protein does and its conservation degree, we compared the number of interactions of the proteins of our networks against the nucleotide diversity of the genes that encode them (Figure 3). Based on this analysis, as the proteins increase the number of interactions that they participate in, their diversity degree, measured here by π (nucleotide diversity index), decrease. From the results obtained for the three predicted networks we can suggest the existence of an evolutionary pressure for the maintenance of a lower diversity in proteins with a high number of interactions.

### 4– Characterizing Modules

At this point, the algorithm networkBLAST was used to identify the modules in the PPIs. The number of conserved modules shared by the three species of *Leishmania* was 199. Despite over millions years of proposed divergence for the analyzed species, this result is not surprising considering that a high synteny was already observed and reported between all sequenced *Leishmania* species [59].

Subsequently, as detailed in Methods section, a function annotation assignment to the network modules was performed using the Biological Process hierarchy of the Gene Ontology and the Perl programming modules GO::TermFinder. This approach allowed the identification of 153 modules which had GO terms with a frequency higher than the expected. In that cases where a given network module received more than one GO term, the most significant one characterized by the smallest P-value in the enrichment analysis was chosen. A complete description including the results obtained for all 153 networks modules annotated can be found in the Table S4. It worths to mention that differently from standard clustering algorithms the networkBLAST approach can produce overlapping modules which makes sense from the biological point of view since one protein can belong to more than one network module.

It is also important to highlight that only 57 unique GO terms were used to describe the 153 network modules predicted and the most frequent terms were assigned to modules which are likely involved in biological processes related to protein folding, translation, tRNA aminoacylation for protein translation, energy derivation by oxidation of organic compounds and carbohydrate metabolism.

Taking into consideration the biological significance of this functional analysis, these results were overlapped with topological analysis.

### 5– Topological Analysis

According to our proposed methodology, two topological indexes (Degree and MCC) were utilized to study the interaction networks predicted here (Table S5). Then, we sorted the proteins present in the PPIs using the MCC index, and we obtained a list of proteins that are central for different cliques (subgraphs) and with high interaction degree (Table S6).

The following analyses were conducted for that list of proteins: a) amino acid variability present in orthologs groups; b) degree of conservation against proteins of three potential hosts (*M. musculus*, *C. lupus familiaris* and *H. sapiens*); and c) epitope computational prediction.

Regarding the variability of these proteins, our results revealed an average identity of 80% between the top 20 proteins ranked by MCC index and their orthologs. Therefore, it was possible to notice that these proteins were relatively conserved among the Kinetoplastids.

On the other hand, only two proteins, LbrM22_V2.0510 (proteasome regulatory ATPase subunit 1) and LmjF36.1650 (proteasome beta 5 subunit), from *L. braziliensis* and *L.major* respectively, had identity higher than 60% when compared against the host proteomes. In addition, *L. infantum* presented 2 proteins with identity higher than 60%, they are LinJ36_V3.1730 (proteasome beta 5 subunit) and LinJ22_V3.0490 (proteasome regulatory ATPase subunit 5).

In this context, we can suggest that the low identity presented by the great majority of the top ranked proteins can be interesting for drug and vaccine studies.

The rationale in suggesting that these proteins could be used for medical purposes can be reinforced by the predicted function of the network modules that they are inserted in. We noted that the most of modules were involved in protein turnover which is known to be involved in responses to vaccination [60]. In addition, we found a total of 9 GO terms describing these modules and 7 of them are shared by the top 20 ranked proteins of each predicted PPI network.

Finally, in respect to immunological potential for these proteins, all of them had more than 5 epitopes predicted for B cells receptors. For the epitope prediction for MHC class I, 12 different alleles were tested and all tested proteins had at minimum of 2 predicted epitopes with potential binding affinity to at least 11 alleles. The last analysis was for epitope predictions of MHC class II. The predictor used for this analysis provides along with the epitope prediction a measure of binding affinity between the epitope and the receptor, and this measure is divided in two categories: weak binding (WB) and strong binding (SB). We selected just predictions which were categorized as SB. Thus, all proteins have at least 2 epitopes with binding affinity to at minimum of 1 allele tested. The total of tested alleles was 17.

### 6– Annotation Prediction for Hypothetical Proteins

In order to address the usefulness of the predicted network to assign some level of functional annotation to hypothetical proteins, we decided to use an approach called FS-Weight that takes into account both direct and indirect neighbors as detailed at Method section. From the total number of proteins covered by the networks, approximately 21% were originally annotated as hypothetical. From this set of proteins, nearly 40%, 48%, and 55% of them received some GO term based on the FS-Weight approach (Table 4). In addition, it is important to point out that this approach provides a score for all annotation prediction that ranges from 0 to 1, and that just GO terms which received a score equal to 1 were considered. Furthermore, when we crossed the information on modules against the hypothetical proteins that received a putative function it was possible to note that for the three networks the hypothetical proteins are more frequently present in modules involved in RNA metabolism. All proteins that received a functional annotation are available in Table S7.

## Discussion

Based on the results obtained regarding the accuracy of the proposed approach for network prediction (AUC value equal to 0.94), we can state that the prediction methodology is relatively reliable (Figure 1), and the predicted protein interactions own a good confidence. However, as it was said on Results section, the databases used for the methodology have many interactions of *E. coli*. This might make the performance evaluation a practice of circular reasoning, and thus lead it to some degree of bias.

In addition, we compared our interaction score schema against others previously published [61]. It is clear from the obtained results (AUC values presented in Table 1) that the score schema we used outperformed the others. Therefore, we applied our interaction score schema for leishmania PPI networks predictions.

Furthermore, the lost associated with the filtering step (detailed in results) was small and this result reflects the quality of genome annotation for the three different leishmanias, which is valuable since our main input data was the protein sequences and the final results depended on the quality of them.

Still on the computational prediction quality issue, in our results we described the assessment of the PPIs based on some known network models such as scale-free model [9] to guarantee their confidence. It is possible to suggest that the PPI networks predicted are consistent as they present features which are common for biological networks currently described. In addition, when the PPIs were compared to random networks (Table 3), it was possible to notice that the values of the Clustering Coefficient of the PPIs are much greater than the random networks, a find that once again suggests the PPIs prediction strength and the absence of spurious interactions. Both results can be used to illustrate the confidence of interolog mapping approach and to reinforce the result found for its evaluation performance, even when there might be a possibility of bias on the evaluation.

In terms of the number of proteins present in the PPIs, our findings are comparable to those found for *L. major* by Flórez 2010, which found nearly 16% of the *L. major* predicted proteome in a predicted PPI. According to the authors, the reason for the small number of proteins mapped in PPIs is a reflection of low levels of similarity between leishmania species and the used database content. On the other hand, the differences between the predicted number of interactions observed in our work and Flórez 2010 can be explained by the different sources of information and approaches used.

Following the network assessment, the first analysis performed in the three PPIs was a Gene Ontology functional annotation. Moreover, it is also noteworthy that the most frequent terms for the three networks regarding Molecular Function ontology are related with binding function, which makes sense since the proteins present in the PPIs are predicted to interact with each other. On the other hand, about the Cellular Component category, we observed terms associated with protein complexes such as proteasome and ribosome. Again, this was somehow expected since a set of interacting proteins possibly are going to form complexes. However, for Biological Process, we obtained a higher diversity of terms that can be hypothesized to be explained by the fact that the same protein can participate in many different processes in a cell.

We also performed an evolution analysis in order to verify whether there was any trend related to the number of interactions and protein sequence diversity. Our results indicate that the number of interactions and diversity are inversely proportional, meaning that as the diversity increases, the number of interactions decreases. In protein-protein interaction networks, proteins presenting several interactions (high degree) are generally called *hub* nodes and genome-wide studies [62], [63] have shown that the deletion of a hub protein is more likely to be lethal than the deletion of a non-hub protein (centrality-lethality rule). In addition, this finding makes sense because these proteins probably are involved in different biological process within a cell with relative success and, in this context, if a random mutation happens, it will likely produce a negative outcome.

Therefore, the points raised herein show that the predicted PPI networks are biologically consistent. Otherwise, we had a trend of proteins with a wide diversity of conservation as hubs.

Another point addressed in our analysis was the network modularity of the predicted PPIs. Modularity is one measure of the structure of networks and many previous works have reported that biological networks are modular [10], [37], [43], [64]. This feature is important for their robustness since a modular architecture guarantees that a system failure is isolated [37]. Thus, if we are interested in destabilizing the PPIs for drug or vaccine purposes, we need to know the modules present in the networks.

In this context, aiming to measure modularity, a clustering analysis was performed in order to identify conserved modules. As it was stated in results section, we found 153 conserved modules which had a function assigned by the enrichment analysis, and these modules can be grouped in 57 different functions.

Based on these findings, it is possible to note that there are many protein complexes (modules) that are essential for the studied organisms. Thus, it is worth to explore in more details these complexes along with the topological information of the network proteins with the potential to elect new potential proteins targets for vaccine and drug development.

In addition, other sources of information were integrated to topological analysis, such as immunological potential, degree of protein sequence conservation among orthologs and degree of identity compared to proteins of potential parasite hosts (human, dog and mouse).This information integration provides a better understanding that can be valuable to select new potential biological targets.

Using this rationale, we suggested a list of proteins (Table S6) that can be attractive for medical purposes. These proteins have a low identity against proteins from hosts, they are potentially recognized by B cells and T cell receptors and are highly conserved compared to their orthologs. In addition, they seem to be central for many biological processes as they have high values of MCC and degree indexes, thus if they are neutralized all the system of protein interaction might suffer severe damage.

Moreover, those proteins do not have high level of identity against the proteins from host proteomes, a desirable characteristic for proteins that will be selected for vaccine development and/or drug therapy. Consequently, side effects can be avoided. Other important feature is the high level of conservation of them when compared against their orthologs; this can guarantee a wide spectrum of action. In the end, they have several potential epitopes which are fundamental for the most important kinds of immunological responses.

Finally, we are interested in using the PPI network information in an annotation framework to assign a putative function to the currently predicted hypothetical proteins. Within the Trypanosamatids context, the study of hypothetical proteins has huge importance, since some organisms, which comprise a part of this group such as the ones that are targets of this work, have around 60% of their predicted proteomes composed of uncharacterized proteins [56], [57]. This scenario is kept current even within the ‘omics’ age because the majority of studies often focus on already well understood and established molecular scenarios. Therefore, the opportunity to expand knowledge further than the known and expected is rarely attempted [65].

Furthermore, the majority of researchers are not interested in investigating the molecular data that are hard to interpret in the light of current biological knowledge, i.e. data on hypothetical proteins [65]. However, the Systems Biology approaches can help to improve these numbers. Thus, there is a group of methods in the Systems Biology context that aims at exploiting information derived from networks to elucidate functional prediction. Hence, various classification methods allow for general function predictions utilizing ‘homology-free’ protein sequence features [65].

An example of the application of a network study to elucidate a function of an uncharacterized protein can be found in the work of Cui *et al* where they built a protein-protein interaction network for *Mycobacterium tuberculosis* using an homology protein mapping approach [66]. In this study, a hypothetical protein with a high degree of interaction was found and evidence for its function came from the fact that it interacts with the same group of ABC transporter ATPase subunits as does a known protein [66]. Thus, this rationale of assigning a function based on the neighbors of a protein can be extremely useful.

In our results, around 50% of the hypothetical proteins present in the networks received some functional annotation. Moreover, the most frequent modules, where those proteins are present, are related to RNA metabolism. This could be interesting as there is currently a huge amount of studies involving different types of RNA and their roles in distinct biological phenomena.

Finally, our group is involved in analysis concerning “Intrinsically Unstructured Proteins” (IUPs) and our previous results (still unpublished) link many features of this group of proteins with the group of hypothetical proteins in Trypanosomatids (data not published). This should be investigated in the future since there are many articles showing how important the IUPs are for the protein-protein interaction networks [67]–[70].

### Conclusion

This work was the first to predict three protein-protein interaction networks for three different species of *Leishmania* and to compare them to each other. A new interaction score schema was proposed and proved to be reliable. Using this strategy, we observed that it is possible to extract important information related to the biology of the studied organism. In addition, using the topological information, we can select proteins that are potential targets for drugs and vaccine development. However, since vaccine and drug prediction represent a complex and multifactorial problem, more data, such as structural data, expression data, etc could be added in order to choose the proteins for future studies in a more efficient way.

In addition, addressing the network information, it was possible to infer some clues regarding some hypothetical proteins that did not have any information related to their molecular functions in the cell.

In summary, based on the evidences reported here, we believe that the networks modeled are biologically consistent and can be useful as tool for different kinds of studies on these organisms.

## Supporting Information

Table S1 ***Leishmania braziliensis*** **PPI network.** Description of all predicted protein interactions and their confidence scores for *L. braziliensis* predicted proteome.
(TXT)

Table S2 ***Leishmania major*** **PPI network.** Description of all predicted protein interactions and their confidence scores for *L. major* predicted proteome.
(TXT)

Table S3 ***Leishmania infantum*** **PPI network.** Description of all predicted protein interactions and their confidence scores for *L. infantum* predicted proteome.
(TXT)

Table S4 **Annotation of Functional Modules (Clusters) predicted for the three PPI networks.** Description of predicted modules including their scores, *p-values* and GO term id.
(TXT)

Table S5 **Topological analysis.** Values of MCC and *Degree* indexes calculated for the proteins present in the three PPI networks modeled.
(TXT)

Table S6 **Analysis of top 20 ranked proteins of each PPI network.** Detailed description of top 20 ranked proteins by MCC index including product description, MCC and *Degree* values, host protein analysis, orthologs analysis and immunological analysis.
(XLS)

Table S7 **Annotation of hypothetical proteins.** Predicted annotation based on FS-Weight and GO ontology assigned to hypothetical proteins present in the networks.
(TXT)
